# Combined Utility of Speckle Tracking Echocardiography and Cardiac Biomarkers for Early Detection of Anthracycline-Induced Cardiotoxicity in Pediatric Oncology Patients

**DOI:** 10.3390/biomedicines12122849

**Published:** 2024-12-14

**Authors:** Cristiana Stolojanu, Ruxandra Steflea, Andrada Mara Micsescu-Olah, Ioana Alexandra, Anca Popoiu, Gabriela Doros

**Affiliations:** 1 Department of Pediatrics, “Victor Babes” University of Medicine and Pharmacy, Eftimie Murgu Square 2, 300041 Timisoara, Romania; cristiana.stolojanu@umft.ro (C.S.); andrada.micsescu-olah@umft.ro (A.M.M.-O.); alexandra.ioana@umft.ro (I.A.); apopoiu@umft.ro (A.P.); doros.gabriela@umft.ro (G.D.); 2 Louis Turcanu Emergency Hospital for Children, Iosif Nemoianu Street 2, 300011 Timisoara, Romania

**Keywords:** pediatric oncology, anthracycline cardiotoxicity, speckle tracking echocardiography, cardiac biomarkers, early detection

## Abstract

Background and Objectives: Anthracycline chemotherapy is a cornerstone in pediatric oncology but carries a significant risk of cardiotoxicity. The early detection of cardiac dysfunction is crucial for timely intervention. This study aims to evaluate the predictive value of combining speckle tracking echocardiography (STE) parameters with traditional cardiac biomarkers for the early detection of anthracycline-induced cardiotoxicity in pediatric oncology patients. Methods: A retrospective cohort study was conducted, involving 99 pediatric oncology patients undergoing anthracycline therapy and 50 age- and sex-matched healthy controls. Cardiac function was assessed using STE parameters—global longitudinal strain (GLS), Simpson’s method of disk ejection fraction (SMOD EF), and myocardial performance index (MPI)—alongside biomarkers including cardiac troponin I (cTnI) and B-type natriuretic peptide (BNP). Assessments were performed at baseline and at 3, 6, and 12 months post-therapy initiation. Results: A total of 28.3% of patients developed cardiotoxicity based on the LVEF decrease. Significant differences were observed between oncological patients under anthracycline treatment and healthy controls. Patients had reduced GLS (−16.1 ± 4.7% vs. −19.6 ± 5.1%, *p* < 0.001), a lower SMOD EF (55.7 ± 6.3% vs. 60.2 ± 6.0%, *p* < 0.001), and a higher MPI (0.38 ± 0.06 vs. 0.33 ± 0.05, *p* < 0.001). Elevated cTnI levels were found in patients compared to controls (3.1 ± 0.9 ng/mL vs. 1.3 ± 0.6 ng/mL, *p* < 0.001). Regression analysis showed that combining GLS, SMOD EF, MPI, and cTnI levels significantly predicted cardiotoxicity (odds ratio = 7.12, 95% CI: 3.04–12.76, *p* < 0.001). Conclusions: Combining STE parameters with cardiac biomarkers enhances the early detection of anthracycline-induced cardiotoxicity in pediatric oncology patients. This combined assessment may facilitate timely interventions to prevent long-term cardiac complications.

## 1. Introduction

The significant strides in pediatric oncology over recent decades have markedly improved survival rates, turning many previously fatal childhood cancers into treatable conditions [1,2]. Anthracyclines, such as doxorubicin and epirubicin, remain essential components of chemotherapy regimens due to their potent anti-neoplastic effects [3,4,5]. However, their known cardiotoxic potential poses a substantial risk, particularly in pediatric patients who may experience long-term cardiac complications [6,7]. Epidemiological studies leveraging large registries and electronic health records indicate that approximately 10–20% of pediatric cancer survivors experience cardiotoxic effects, primarily linked to anthracycline chemotherapy and radiation therapy [8].

Traditional methods for monitoring cardiac function, such as left ventricular ejection fraction (LVEF) assessment via conventional echocardiography, often lack sensitivity for detecting early myocardial changes [9,10]. By the time a decrease in LVEF is apparent, significant and possibly irreversible myocardial damage may have occurred [11,12,13]. This limitation underscores the urgent need for more sensitive diagnostic tools capable of identifying subclinical cardiac dysfunction.

Speckle tracking echocardiography (STE) has emerged as a valuable technique for evaluating myocardial deformation, offering superior sensitivity in detecting subtle changes in cardiac function [14,15,16]. Parameters like global longitudinal strain (GLS) have been shown to identify myocardial impairment earlier than conventional echocardiographic measures [17,18]. Additionally, the myocardial performance index (MPI) and ejection fraction calculated using Simpson’s method of disks (SMOD EF) provide comprehensive assessments of both systolic and diastolic function [19,20].

Cardiac biomarkers, including cardiac troponin I (cTnI) and B-type natriuretic peptide (BNP), serve as biochemical indicators of myocardial injury and stress [21,22]. Elevated levels of these biomarkers have been associated with chemotherapy-induced cardiotoxicity, reflecting ongoing myocardial damage even before functional impairment is evident [23]. However, relying solely on biomarkers may not provide a complete picture of cardiac health.

Integrating STE parameters with cardiac biomarkers could enhance the early detection of cardiotoxicity, enabling timely interventions. This study aims to evaluate the combined utility of STE and cardiac biomarkers in predicting anthracycline-induced cardiotoxicity in pediatric oncology patients, potentially improving long-term cardiac outcomes.

## 2. Materials and Methods

### 2.1. Legal and Ethical Considerations

This retrospective cohort study was conducted at the “Louis Turcanu” Emergency Children’s Hospital affiliated with the “Victor Babes” University of Medicine and Pharmacy. The study received approval from the Institutional Review Board (IRB), ensuring compliance with the ethical standards of the Declaration of Helsinki and its amendments. Written informed consent was obtained from the parents or legal guardians of all participants, and assent was obtained from children aged seven years and older when appropriate.

### 2.2. Inclusion and Exclusion Criteria

We included pediatric patients aged 1 to 18 years who were newly diagnosed with cancer and scheduled to receive anthracycline-based chemotherapy. The inclusion criteria required patients to have a new diagnosis of a pediatric malignancy necessitating anthracycline therapy; no pre-existing cardiac diseases, congenital heart defects, or significant comorbidities affecting cardiac function; and no prior exposure to cardiotoxic agents or radiotherapy. It was also essential that baseline echocardiographic assessments and cardiac biomarker measurements were available before the initiation of chemotherapy, along with follow-up data at 3, 6, and 12 months post-therapy initiation.

Exclusion criteria for the study included patients with known cardiac pathology or significant comorbidities affecting cardiac function; those with prior chemotherapy or radiotherapy exposure, incomplete medical records, or loss to follow-up during the study period; and those who refused or were unable to provide informed consent. To establish a baseline comparison, a control group of 50 age- and sex-matched healthy children was recruited from outpatient pediatric clinics. These controls had no history of cardiac disease, normal physical examinations, and normal baseline echocardiographic and cardiac biomarker assessments. In this study, none of the patients received prior exposure to cardiotoxic agents or radiotherapy. All those who received such were excluded from the analysis, thereby eliminating potential confounding factors and enhancing the validity of our findings regarding cardiotoxicity in pediatric oncology patients.

### 2.3. Measurements and Definitions

Patients received anthracycline chemotherapy as part of standard pediatric oncology protocols appropriate for their cancer type. The anthracyclines administered included doxorubicin and epirubicin, with dosages adjusted according to body surface area (BSA) and specific treatment regimens. Cumulative anthracycline doses were recorded for each patient. Chemotherapy regimens also included other agents such as cyclophosphamide, vincristine, and methotrexate, depending on the specific protocol and cancer type.

Echocardiographic evaluations were performed using a GE Vivid E9 (Boston, MA, USA) ultrasound machine equipped with a 3.5 MHz transducer. Baseline echocardiograms were conducted within one week before chemotherapy initiation, with follow-up assessments at 3, 6, and 12 months post-therapy initiation.

Speckle tracking echocardiography (STE) was utilized to assess myocardial deformation. Global longitudinal strain (GLS) was measured using semi-automated software, analyzing apical four-chamber, two-chamber, and three-chamber views. The frame rate was set between 50 and 80 frames per second to optimize speckle tracking accuracy. Adequate image quality was ensured by adjusting gain settings and avoiding foreshortening of the ventricle. Manual adjustments were made when necessary to define the endocardial borders accurately.

Left ventricular ejection fraction (LVEF) was calculated using standard mode ejection fraction (SMOD) from biplane apical views. The myocardial performance index (MPI) was determined using pulsed-wave Doppler recordings of mitral inflow and left ventricular outflow tract velocities, providing an index of combined systolic and diastolic function. The MPI, an essential measure of both systolic and diastolic cardiac functions, was calculated using the formula MPI = (IVCT + IVRT)/ET, where IVCT (Isovolumetric Contraction Time) is the interval from the closure of the mitral valve to the opening of the aortic valve, IVRT (Isovolumetric Relaxation Time) is the interval from the closure of the aortic valve to the opening of the mitral valve, and ET (Ejection Time) is the duration of blood ejection from the left ventricle through the aortic valve.

Two experienced pediatric cardiologists, blinded to the patients’ clinical data, independently reviewed the echocardiograms. Inter-observer variability was assessed using intraclass correlation coefficients, and discrepancies were resolved by consensus.

Blood samples were collected for cardiac biomarker analysis at the same time points as echocardiographic assessments. Cardiac troponin I (cTnI) levels were measured using a high-sensitivity immunoassay (Abbott Architect STAT, Abbott Laboratories, Hoofddorp, The Netherlands), with a lower detection limit of 0.01 ng/mL. B-type natriuretic peptide (BNP) levels were measured using a chemiluminescent immunoassay (ADVIA Centaur BNP Assay, Siemens Healthineers, Erlangen, Germany), with a detection range of 0–5000 pg/mL. A reduction in GLS greater than 15% from baseline and levels of cTnI (>0.04 ng/mL) were considered abnormal. All assays were performed in the hospital’s central laboratory, adhering to standard operating procedures and quality control measures.

Cardiotoxicity was determined based on the guidelines from the American Society of Echocardiography and the European Association of Cardiovascular Imaging, identified by a decrease in LVEF of 10% or more from baseline to a value below the normal lower limit (<50%).

### 2.4. Data Collection and Management

Demographic data, including age, sex, height, weight, and BMI, were collected from medical records. Clinical data included cancer type, treatment protocol, cumulative anthracycline dose, and use of cardioprotective agents. All data were anonymized and entered into a secure database for analysis. Data integrity was ensured through double-entry verification and regular audits.

### 2.5. Statistical Analysis

Statistical analyses were performed using SPSS version 26.0 (IBM Corp., Armonk, NY, USA). Continuous variables were expressed as mean ± standard deviation (SD) or median with interquartile range (IQR), depending on data distribution assessed by the Shapiro–Wilk test. Categorical variables were presented as counts and percentages.

Comparisons between groups were conducted using independent *t*-tests for normally distributed continuous variables and Mann–Whitney U tests for non-normally distributed variables. Chi-square tests or Fisher’s exact tests were used for categorical variables. Repeated measures ANOVA or Friedman tests were used to assess changes in echocardiographic parameters and biomarkers over time within groups, with Bonferroni correction applied for multiple comparisons.

Pearson’s or Spearman’s correlation coefficients were calculated to assess associations between echocardiographic parameters and biomarkers. Multivariate logistic regression analysis was performed to identify independent predictors of cardiotoxicity, adjusting for potential confounders such as age, sex, BMI, and cumulative anthracycline dose. The multivariate logistic regression analysis for predicting cardiotoxicity utilized the post-treatment (12-month) values of parameters such as GLS, SMOD EF, MPI, cTnI, and BNP. The model’s goodness-of-fit was assessed using the Hosmer–Lemeshow test. *p*-values less than 0.05 were considered statistically significant.

## 3. Results

### 3.1. Participant Characteristics

The two groups were well matched, with no significant difference in terms of age, sex, BMI, or BMI category (Table 1). Acute Lymphoblastic Leukemia was the most common malignancy, representing 31.3% of the cases, with patients receiving an average Doxorubicin dose of 282 ± 71 mg/m^2^. Hodgkin Lymphoma accounted for 14.1% of cases, treated with slightly higher Doxorubicin doses averaging 319 ± 82 mg/m^2^. Osteosarcoma patients, comprising 12.1% of the cohort, received the highest average Doxorubicin dose at 448 ± 52 mg/m^2^, indicating a possibly more intensive treatment protocol. Neuroblastoma and Ewing Sarcoma were less common, making up 10.1% and 9.1% of the cases, respectively, with Doxorubicin doses of 301 ± 63 mg/m^2^ and 352 ± 91 mg/m^2^. Rhabdomyosarcoma and Wilms Tumor each constituted 8.1% of cases, but these patients were treated with Epirubicin, with average doses of 279 ± 66 mg/m^2^ and 263 ± 54 mg/m^2^, respectively. A small subset labeled as “Others” comprised 7.1% of the cohort and received a mix of Doxorubicin and Epirubicin, with a mean dose of 309 ± 74 mg/m^2^ (Table 2).

### 3.2. Baseline GLS and Biomarker Levels

The GLS was slightly lower in patients (−18.5 ± 2.3%) compared to controls (−19.0 ± 2.5%), but this difference was not statistically significant (*p* = 0.12). Similarly, the SMOD EF showed minimal difference between the two groups, with patients at 60.5 ± 5.2% and controls at 61.0 ± 5.1% (*p* = 0.48). The MPI was identical in both cohorts (0.33 ± 0.04, *p* = 0.85). Cardiac biomarkers, including cTnI and BNP, were slightly higher in patients (cTnI: 0.02 ± 0.01 ng/mL; BNP: 35 ± 10 pg/mL) compared to controls (cTnI: 0.01 ± 0.01 ng/mL; BNP: 32 ± 9 pg/mL), yet these differences were also not significant (cTnI *p* = 0.06; BNP *p* = 0.14), as seen in Table 3.

At baseline, the GLS was recorded at −18.5 ± 2.3%, which gradually worsened to −16.0 ± 3.1% by the 12-month follow-up. Similarly, the SMOD EF showed a progressive decline from an initial 60.5 ± 5.2% to 54.8 ± 6.5% at the end of the year. The MPI also increased from 0.33 ± 0.04 to 0.40 ± 0.05, indicating a worsening in myocardial efficiency, as presented in Table 4.

Starting from baseline values of cTnI at 0.02 ± 0.01 ng/mL and BNP at 35 ± 10 pg/mL, there was a continuous rise in these biomarkers over the 12-month period. By the 12-month mark, cTnI levels had escalated to 0.08 ± 0.03 ng/mL, and BNP levels had increased to 85 ± 25 pg/mL. The statistical trend analysis revealed a highly significant increase (*p* < 0.001) in both biomarkers (Table 5).

The cardiotoxic group exhibited worse GLS at −14.0 ± 2.5% compared to −17.0 ± 2.7% in the non-cardiotoxic group (*p* < 0.001), a lower SMOD EF of 50.5 ± 4.5% versus 56.8 ± 5.2% (*p* < 0.001), and a higher myocardial performance index (MPI) of 0.43 ± 0.04 compared to 0.38 ± 0.04 (*p* < 0.001). Additionally, cardiac biomarkers like cTnI and BNP were significantly higher in the cardiotoxicity group, with cTnI at 0.12 ± 0.03 ng/mL and BNP at 110 ± 20 pg/mL, versus 0.06 ± 0.02 ng/mL and 70 ± 15 pg/mL, respectively, in the non-cardiotoxic group (*p* < 0.001 for both) (Table 6).

### 3.3. Correlation and Regression Analysis

Specifically, 13.90% of patients receiving doses below 300 mg/m^2^ developed cardiotoxicity, compared to 26.70% in the 300–400 mg/m^2^ group, 37.50% in the 401–500 mg/m^2^ cohort, and a substantial 52.90% of those administered doses exceeding 500 mg/m^2^. Overall, 28.30% of the total patient population experienced cardiotoxicity (Table 7).

**Figure 1:**
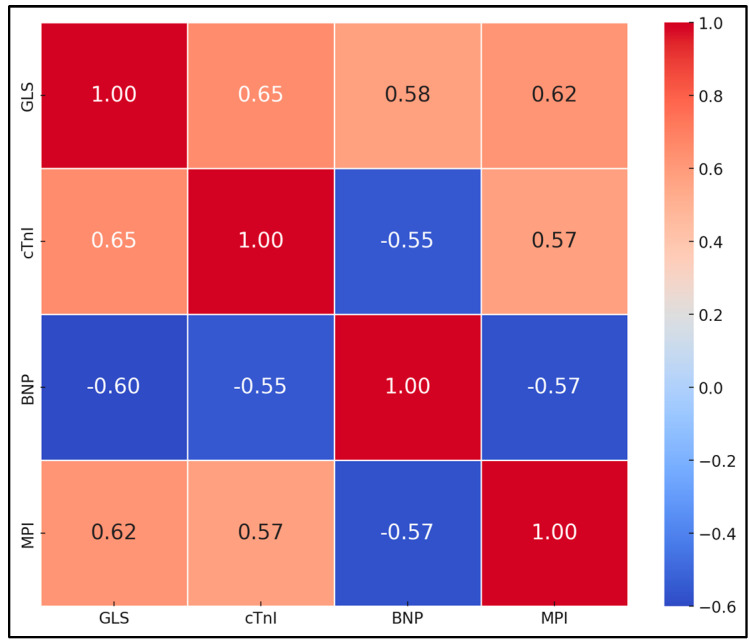
Correlation coefficients between echocardiographic parameters and biomarkers at 12 months.

GLS showed a strong positive correlation with cTnI and BNP, with correlation coefficients of 0.65 and 0.58, respectively, both statistically significant (*p* < 0.001). Similarly, SMOD EF demonstrated negative correlations with cTnI and BNP, at −0.60 and −0.55, respectively (*p* < 0.001), suggesting that decreases in EF are associated with increases in biomarker levels. The MPI also showed positive correlations with cTnI and BNP (0.62 and 0.57, respectively, *p* < 0.001) (Table 8 and Figure 1).

**Figure 2:**
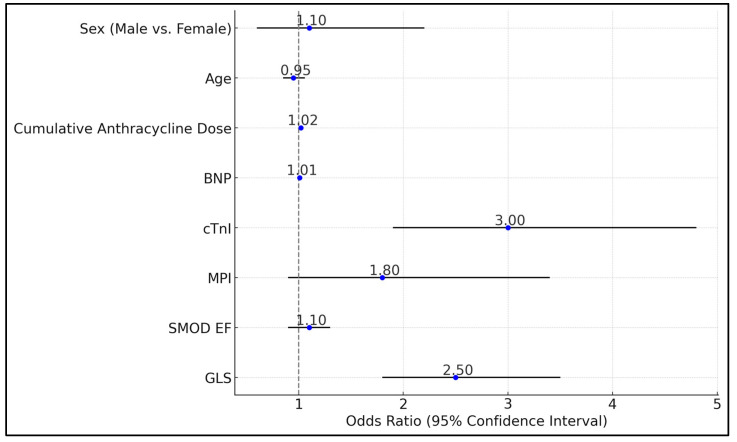
Multivariate logistic regression analysis for predicting cardiotoxicity.

The GLS and cTnI emerged as strong predictors of cardiotoxicity, with GLS showing an odds ratio (OR) of 2.5 (95% CI: 1.8–3.5, *p* < 0.001) and cTnI having an OR of 3 (95% CI: 1.9–4.8, *p* < 0.001). Additionally, the cumulative anthracycline dose received by patients was significantly associated with an increased risk of cardiotoxicity, with an OR of 1.02 per mg/m^2^; increase (95% CI: 1.01–1.03, *p* = 0.002). In contrast, the standard mode ejection fraction (SMOD EF), MPI, BNP, age, and sex did not show significant associations with cardiotoxicity in this analysis, with *p*-values indicating a lack of statistical significance (SMOD EF: *p* = 0.2, MPI: *p* = 0.08, BNP: *p* = 0.12, Age: *p* = 0.35, Sex: *p* = 0.75), as seen in Table 9 and Figure 2.

## 4. Discussion

### 4.1. Analysis of Findings

This study demonstrates that combining STE parameters with cardiac biomarkers significantly improves early detection of anthracycline-induced cardiotoxicity in pediatric oncology patients. The progressive deterioration in GLS, SMOD EF, and MPI over time highlights the cumulative cardiotoxic effect of anthracyclines. The significant correlations between echocardiographic parameters and biomarkers suggest a synergistic role in detecting myocardial injury. Elevated cTnI and BNP levels were associated with worsening GLS and SMOD EF, indicating that biochemical markers of myocardial injury correspond with functional impairment.

In a similar manner to how the study by Jefferies et al. [24] focused on the long-term cardiac impacts of anthracycline and radiotherapy in childhood cancer survivors, research by Ardelean et al. [25] evaluated the immediate effects of anthracycline dosages on pediatric hemato-oncology patients using speckle tracking echocardiography. Jefferies et al. reported that 28.2% of survivors exhibited concentric remodeling and those exposed to chest radiotherapy demonstrated a higher propensity (41%) compared to those exposed only to anthracyclines (24%), with a significant association between concentric remodeling and decreased exercise tolerance (odds ratio, 1.75; 95% CI, 1.15–2.68). On the other hand, Ardelean et al. found that higher doses of Doxorubicin were associated with increased cardiotoxicity, as indicated by the negative correlation between the anthracycline dose and global longitudinal strain (Rho = −0.411, *p* = 0.001). While Jefferies et al. identified a clear link between therapy type and geometric changes in the heart, Ardelean et al. suggested that despite STE’s sensitivity in detecting early myocardial injury, traditional biomarkers like Troponin I and B-type natriuretic peptide remain crucial for a comprehensive assessment, given that STE parameters did not independently predict cardiotoxicity.

Similarly, the study by Yu Kang et al. [26] explored the early detection and prediction of cardiotoxicity during epirubicine-based chemotherapy in adult patients with non-Hodgkin lymphoma, focusing on speckle tracking echocardiography combined with high-sensitivity cardiac troponin T. They found significant decreases in myocardial strain values and an increase in cTnT levels, where a greater than 15.9% reduction in global longitudinal strain and a cTnT increase of more than 0.004 ng/mL from baseline to the third chemotherapy cycle were predictive of later cardiotoxicity, with GLS demonstrating high sensitivity (86%) and specificity (75%). Conversely, the cross-sectional study by Daniel A. Mulrooney et al. [27] assessed long-term cardiac outcomes in adult survivors of childhood cancer, revealing a prevalence of cardiomyopathy in 7.4% of survivors, with higher incidences correlated with increased doses of anthracycline and radiation exposure.

In their observational study, Avilès et al. [28] utilized speckle tracking echocardiography to assess cardiac toxicity in children exposed to anthracyclines during fetal development and found no significant cardiac abnormalities when compared to controls, suggesting the safety of anthracycline use during pregnancy with respect to cardiac outcomes in the children. In a similar manner, the study by Pascal Amedro et al. [29] also employed STE but focused on detecting late anthracycline-induced cardiotoxicity in children after cancer remission. Amedro’s findings indicated significant alterations in left ventricular strain patterns, with a decreased global longitudinal strain of −19.1% in the anthracycline group versus −21.5% in controls and a higher incidence of abnormal GLS values (18.6% vs. 1.0%, *p* < 0.0001), despite normal conventional echocardiographic measures. These results suggest that while Avilès et al. did not observe early cardiac changes post-anthracycline exposure during pregnancy, Amedro et al. documented sub-clinical cardiac dysfunction years after treatment, highlighting the need for long-term cardiac monitoring in pediatric patients treated with anthracyclines.

Similarly, Özlem Arman Bilir et al. [30] explored the cardiotoxic effects of anthracyclines in children with acute lymphoblastic leukemia (ALL), using speckle tracking echocardiography and tissue Doppler imaging (TDI). The study found that the myocardial velocity during systole (Sm) at the interventricular septum significantly decreased from baseline to the end of the induction phase, emphasizing the onset of cardiotoxicity. Specifically, the global longitudinal strain rate showed a marked reduction in both left and right ventricles, indicating early cardiac changes. Similarly, Vivian Wing-Yi Li et al. [31] conducted a systematic review and meta-analysis encompassing 42 studies with a total of 5430 children, examining myocardial deformation via STE. Their findings indicated that left ventricular systolic deformation was consistently impaired during initial treatment and in long-term childhood cancer survivors (CCSs), with a pooled analysis showing significant strain reductions. However, data on right ventricular deformation were limited and inconclusive

Philip T. Levy et al. [32] conducted a systematic review and meta-analysis to establish reference ranges for left ventricular strain measures in children using two-dimensional speckle tracking echocardiography (2DSTE), analyzing data from 2325 children across 43 datasets. They reported a normal mean global longitudinal strain of −20.2%, global circumferential strain (GCS) of −22.3%, and global radial strain (GRS) of 45.2%. In a similar manner, the study by Süha Çetin et al. [33] evaluated subclinical cardiotoxicity in childhood cancer survivors who had been treated with anthracyclines, finding reduced longitudinal and radial strain values compared to controls, despite normal ejection fraction and fractional shortening.

Moreover, in order to have an early identification of cardiac toxicity, the recent literature underscores the significant role of high-sensitivity C-reactive protein (hsCRP) as a robust biomarker of systemic inflammation, which is increasingly recognized as a pivotal biomarker for inflammation and cardiac injury, particularly in the context of chemotherapy-induced cardiotoxicity and ischemic heart disease. In the study by Hasan et al. [34], elevated pre-treatment hsCRP levels were identified as a robust prognostic marker for cardiotoxicity in breast cancer patients undergoing anthracycline-based chemotherapy regimens such as AC and AC→T. The research demonstrated that patients receiving more intensive chemotherapy combinations exhibited significantly higher hsCRP levels, which correlated with the severity and stage of cancer, underscoring hsCRP’s role in predicting cardiac side effects. Concurrently, Tong et al. [35] found a strong positive correlation between hsCRP levels and echocardiographic indicators of coronary microvascular dysfunction (MVD) in patients with ischemic heart disease. Their findings revealed that elevated hsCRP was a significant predictor of severe MVD, which is associated with myocardial injury, thereby highlighting the integral relationship between systemic inflammation and echocardiographic measures of cardiac function. These studies collectively emphasize that hsCRP not only serves as a marker of systemic inflammation but also closely correlates with echocardiographic findings, enhancing its utility in the early detection and monitoring of cardiac dysfunction.

Emerging biomarkers such as Interleukin-6 (IL-6), Interleukin-37 (IL-37), and Galectin-3 (Gal-3) are gaining attention for their potential roles in evaluating cardiac toxicity. IL-6, a cytokine involved in inflammation, has been studied for its ability to signal cardiac stress and potential damage. IL-37, known for its anti-inflammatory properties, may play a protective role in cardiovascular diseases by modulating immune responses [36]. Galectin-3, a member of the lectin family, has shown significant potential in understanding cardiac fibrosis and remodeling, conditions closely linked to heart failure and adverse cardiac events. It is specifically implicated in chronic inflammation and myocardial tissue remodeling post-injury, and its levels have been associated with the severity and prognosis in various cardiovascular conditions like heart failure, myocardial infarction, and diastolic dysfunction. These biomarkers are not yet fully integrated into clinical practice but represent a promising direction for early detection and stratification of cardiac risk in patients, particularly those undergoing treatments that may induce cardiotoxicity [37,38,39].

Implementing combined assessments may allow for earlier identification of at-risk patients, facilitating timely interventions such as dose adjustments or cardioprotective therapies. This approach could mitigate long-term cardiac complications, improving quality of life and survival outcomes.

### 4.2. Study Limitations

The retrospective design of this study presents several inherent limitations, including potential biases related to patient selection and data collection. While efforts were made to standardize echocardiographic assessments and biomarker measurements, variability in imaging quality and inter-observer interpretation could affect the accuracy of the results. The use of a single institution for patient recruitment may limit the generalizability of the findings to other settings or populations. Furthermore, although the study aimed to establish correlations between speckle tracking echocardiography (STE) parameters and cardiac biomarkers, the observational nature of the study does not allow for causal inferences. Additionally, while significant findings were reported, the small sample size, especially for subgroup analyses, may limit the statistical power to detect minor but clinically relevant differences. Another limitation is the exclusion of patients with any prior exposure to cardiotoxic treatments or existing cardiac conditions, which could exclude a subset of patients at a higher risk of cardiotoxicity.

## 5. Conclusions

This study concluded that integrating speckle tracking echocardiography parameters with cardiac biomarker levels can enhance the early detection of anthracycline-induced cardiotoxicity in pediatric oncology patients. The combined use of these diagnostic tools allows for the identification of subclinical cardiac changes before conventional echocardiographic indices such as ejection fraction reveal abnormalities. Early detection is crucial for timely intervention, potentially improving long-term cardiac outcomes in this vulnerable population. The findings underscore the value of comprehensive cardiac monitoring protocols in pediatric oncology, incorporating advanced imaging techniques and sensitive biomarkers to assess myocardial function dynamically over the course of chemotherapy. Such protocols can facilitate early therapeutic adjustments, aiming to minimize cardiac damage and improve the quality of life and survival of childhood cancer survivors.

## Figures and Tables

**Table 1:** Demographic and clinical characteristics of study participants.

Variables	Patients (*n* = 99)	Controls (*n* = 50)	*p*
Age (years, mean ± SD)	10.6 ± 4.3	10.8 ± 3.8	0.78
Age range (years)	2–17	3–18	
Sex (Male/Female)	57/42	28/22	0.85
BMI (kg/m^2^, mean ± SD)	19.2 ± 3.7	19.5 ± 3.6	0.68
Underweight (<5th %)	5 (5.1%)	2 (4.0%)	0.75
Normal weight (5th–85th %)	80 (80.8%)	41 (82.0%)	0.88
Overweight (>85th %)	14 (14.1%)	7 (14.0%)	0.99
**Treatment**			
Anthracycline only	21 (21.2%)	N/A	–
Anthracycline + Vincristine	33 (33.3%)	N/A	–
Anthracycline + Methotrexate	14 (14.1%)	N/A	–
Anthracycline + Cyclophosphamide	27 (27.3%)	N/A	–
Multi-agent combination	7 (7.1%)	N/A	–

BMI—Body Mass Index; SD—Standard Deviation.

**Table 2:** Distribution of cancer types and treatment protocols among patients.

Variables	Patients (*n* = 99)	Type of Anthracycline	Dose
Acute Lymphoblastic Leukemia	31 (31.3%)	Doxorubicin	282 ± 71
Hodgkin Lymphoma	14 (14.1%)	Doxorubicin	319 ± 82
Osteosarcoma	12 (12.1%)	Doxorubicin	448 ± 52
Neuroblastoma	10 (10.1%)	Doxorubicin	301 ± 63
Ewing Sarcoma	9 (9.1%)	Doxorubicin	352 ± 91
Rhabdomyosarcoma	8 (8.1%)	Epirubicin	279 ± 66
Wilms Tumor	8 (8.1%)	Epirubicin	263 ± 54
Others	7 (7.1%)	Doxorubicin/Epirubicin	309 ± 74

**Table 3:** Baseline echocardiographic parameters and cardiac biomarker levels.

Parameters	Normal Range	Patients (*n* = 99)	Controls (*n* = 50)	*p*
GLS (% mean ± SD)	−18% to −22%	−18.5 ± 2.3	−19.0 ± 2.5	0.12
SMOD EF (% mean ± SD)	55% to 70%	60.5 ± 5.2	61.0 ± 5.1	0.48
MPI (mean ± SD)	0.25 to 0.45	0.33 ± 0.04	0.33 ± 0.04	0.85
cTnI (ng/mL mean ± SD)	<0.01 ng/mL	0.02 ± 0.01	0.01 ± 0.01	0.06
BNP (pg/mL mean ± SD)	<100 pg/mL	35 ± 10	32 ± 9	0.14

GLS—Global Longitudinal Strain; EF—Ejection Fraction; SMOD EF—Standard Mode Ejection Fraction; MPI—Myocardial Performance Index; cTnI—Cardiac Troponin I; BNP—Brain Natriuretic Peptide.

**Table 4:** Longitudinal changes in echocardiographic parameters in patients.

Time Point	GLS (% Mean ± SD)	SMOD EF (% Mean ± SD)	MPI (Mean ± SD)	*p* (Trend)
Baseline	−18.5 ± 2.3	60.5 ± 5.2	0.33 ± 0.04	
3 Months	−17.5 ± 2.6	58.0 ± 5.6	0.35 ± 0.04	
6 Months	−16.8 ± 2.9	56.2 ± 6.0	0.37 ± 0.05	
12 Months	−16.0 ± 3.1	54.8 ± 6.5	0.40 ± 0.05	<0.001

GLS—Global Longitudinal Strain; EF—Ejection Fraction; SMOD EF—Standard Mode Ejection Fraction; MPI—Myocardial Performance Index; cTnI—Cardiac Troponin I; SD—Standard Deviation.

**Table 5:** Longitudinal changes in cardiac biomarkers in patients.

Time Point	cTnI (ng/mL Mean ± SD)	BNP (pg/mL Mean ± SD)	*p* (Trend)
Baseline	0.02 ± 0.01	35 ± 10	
3 Months	0.04 ± 0.02	50 ± 15	
6 Months	0.06 ± 0.02	65 ± 20	
12 Months	0.08 ± 0.03	85 ± 25	<0.001

cTnI—Cardiac Troponin I; SD—Standard Deviation; BNP—Brain Natriuretic Peptide.

**Table 6:** Comparison of echocardiographic parameters and biomarkers at 12 months.

Parameters	Cardiotoxicity (*n* = 28)	No Cardiotoxicity (*n* = 71)	*p*
GLS (% mean ± SD)	−14.0 ± 2.5	−17.0 ± 2.7	<0.001
SMOD EF (% mean ± SD)	50.5 ± 4.5	56.8 ± 5.2	<0.001
MPI (mean ± SD)	0.43 ± 0.04	0.38 ± 0.04	<0.001
cTnI (ng/mL mean ± SD)	0.12 ± 0.03	0.06 ± 0.02	<0.001
BNP (pg/mL mean ± SD)	110 ± 20	70 ± 15	<0.001

cTnI—Cardiac Troponin I; SD—Standard Deviation; BNP—Brain Natriuretic Peptide; GLS—Global Longitudinal Strain; EF—Ejection Fraction; SMOD EF—Standard Mode Ejection Fraction; MPI—Myocardial Performance Index.

**Table 7:** Relationship between cumulative anthracycline dose and cardiotoxicity in pediatric oncology patients.

Cumulative Anthracycline Dose (mg/m^2^)	Number of Patients (*n* = 99)	Number with Cardiotoxicity (*n* = 28)	Percentage with Cardiotoxicity (%)	Percentage with Clinical Heart Failure (%)
**<300**	36	5	13.90%	2.78%
**300–400**	30	8	26.70%	8.57%
**401–500**	16	6	37.50%	14.29%
**>500**	17	9	52.90%	17.86%
**Total**	**99**	**28**	**28.30%**	**46.43%**

**Table 8:** Correlation coefficients between echocardiographic parameters and biomarkers at 12 months.

Parameters	cTnI Correlation (r)	BNP Correlation (r)	*p*
GLS	0.65	0.58	<0.001
SMOD EF	−0.60	−0.55	<0.001
MPI	0.62	0.57	<0.001

GLS—Global Longitudinal Strain; EF—Ejection Fraction; SMOD EF—Standard Mode Ejection Fraction; MPI—Myocardial Performance Index; AUC—Area Under Curve.

**Table 9:** Multivariate logistic regression analysis for predicting cardiotoxicity.

Variables	Odds Ratio	95% CI	*p*
GLS	2.5	1.8–3.5	<0.001
SMOD EF	1.1	0.9–1.3	0.2
MPI	1.8	0.9–3.4	0.08
cTnI	3	1.9–4.8	<0.001
BNP	1.01	0.99–1.02	0.12
Cumulative Anthracycline Dose (mg/m^2^)	1.02	1.01–1.03	0.002
Age	0.95	0.85–1.06	0.35
Sex (Male vs. Female)	1.1	0.6–2.2	0.75

GLS—Global Longitudinal Strain; EF—Ejection Fraction; SMOD EF—Standard Mode Ejection Fraction; MPI—Myocardial Performance Index; CI—Confidence Interval.

## Data Availability

The data presented in this study are available on request from the corresponding author. The data are not publicly available due to legal and ethical reasons.
